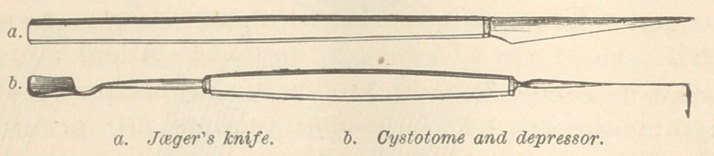# Jæger’s Operation for Cataract

**Published:** 1876-06

**Authors:** Lyman Ware

**Affiliations:** Chicago


### JÆGER'S OPERATION FOR CATARACT.

By LYMAN WARE, M.D., Chicago.

(Read before the Chicago Society of Physicians and Surgeons.)

Prof. Jæger claims the following as some of the advantages of his operation over all others:

1. The possibility of making a complete linear section, thereby facilitating the extraction of the largest lens.
2. The possibility of having complete control over the eye, without fixation while making the section.
3. The *complete retention* of the aqueous humor while the section is being made, which is considered of the *greatest importance.*
4. The proportionally slight increase of the intraocular pressure during the operation.
5. The relatively easy task of removing the lens.

Prof. Jæger says that even among ophthalmologists the line of demarcation between the linear and flap section is not closely drawn. That can only properly be considered a linear section whose entire length is at right angles to the same superficial layer, and which has a

FIG. 2.

FIG. 1.

FIG. 3.

FIG. 4.

FIG. 5.

common axis. In Fig. 1, allow *x x* to represent the several coats of the eye. Then the section *a b c d* in its entire length is in the same direction to the superficial layer, and its breadth is the narrowest possible. In Fig. 2, the knife is not perpendicular to the eye but placed at an angle, and the section *e f g h* is not at right angles to the same superficial surface, and has the greatest possible breadth, and forms consequently, a perfect flap. Figs. 3, 4, and 5, will show the same more clearly. In Fig 3, no portion of the section has the same common axis. Fig. 4 is inclined to a linear section, as that portion of the section *p p* has the same common axis, and is at right angles to the same superficial surface. Fig. 5 is still more inclined, having the portions *o o p p q q* in its entire length in the same angle. Prof. Jæger operates in the sitting posture; is ambidextrous, dispenses altogether with anæsthetics and the eye speculum, depending wholly upon his assistant. The knife he uses is somewhat triangular in form, very similar to Beer's knife, 1½ inches in length, its greatest breadth ⅛ of an inch, its back blunt but very thin; its surface is cylindrically curved, the curve representing a circle with a

*a. Jæger's knife.* *b. Cystotome and depressor.*

radius of ⅛ of an inch. He inserts the knife (the concave surface looking forward) in the outer, upper portion of the sclerotic 2½ or 3 lines from the corneal junction, and 4 or 4½ lines below the upper corneal line; the exit exactly corresponds to the entrance point; the knife is held at an angle of about 45 degrees to the bulb of the eye, and consequently the section lies for the most part in the cornea, and has an average length of about ⅜ of an inch. After iridectomy he opens the capsule with his cystotome, which has a horizontal length of 1⅛ inches, an arm which is at right angles to the shaft and which has a length or projection of ⅛ of an inch, and is provided with a slight hook or projecting needle point. In introducing and removing the cystotome he is particularly careful in keeping it in contact with the posterior corneal surface. In removing the lens he presses lightly with his curette, whose curve corresponds to that of the eye, upon the upper corneal section, producing at the same time counter pressure by means of the index finger placed upon the lower section.

Being ambidextrous, his instruments must of course be duplicated, so as to correspond with the eye being operated upon.

Prof. Jæger's statistics are not yet sufficiently full and complete to judge impartially of his ingenious and long studied operation, yet he is confident that its many excellencies will recommend it favorably to the profession.

## Figures and Tables

**Fig. 1. f1:**
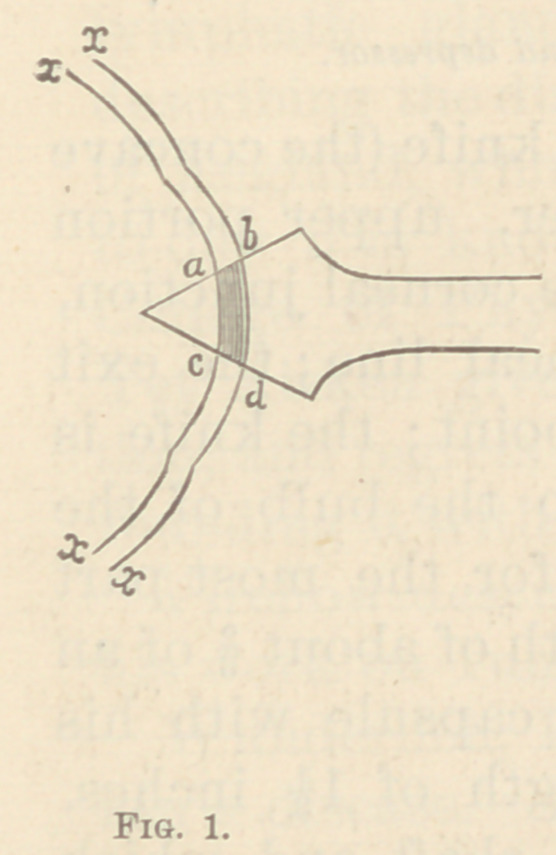


**Fig. 2. f2:**
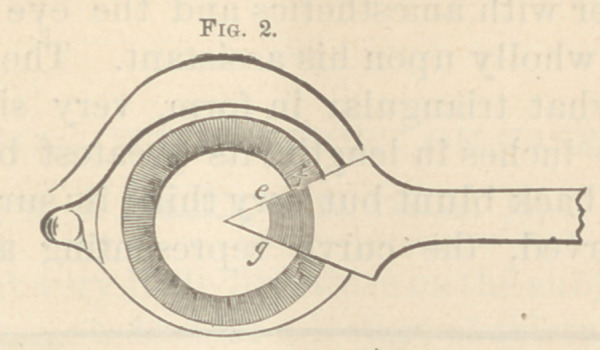


**Fig. 3. f3:**
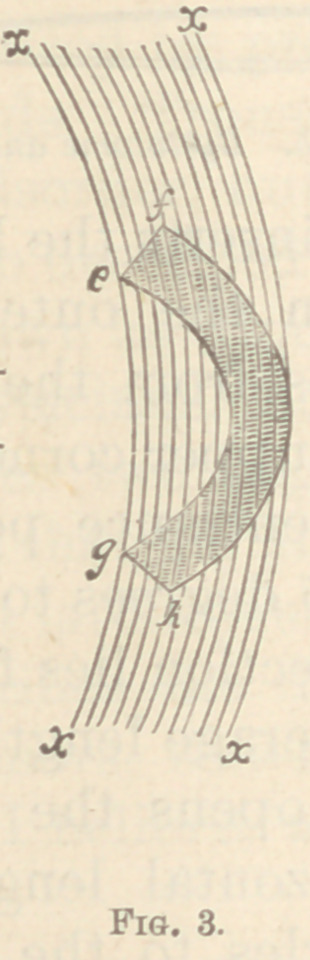


**Fig. 4. f4:**
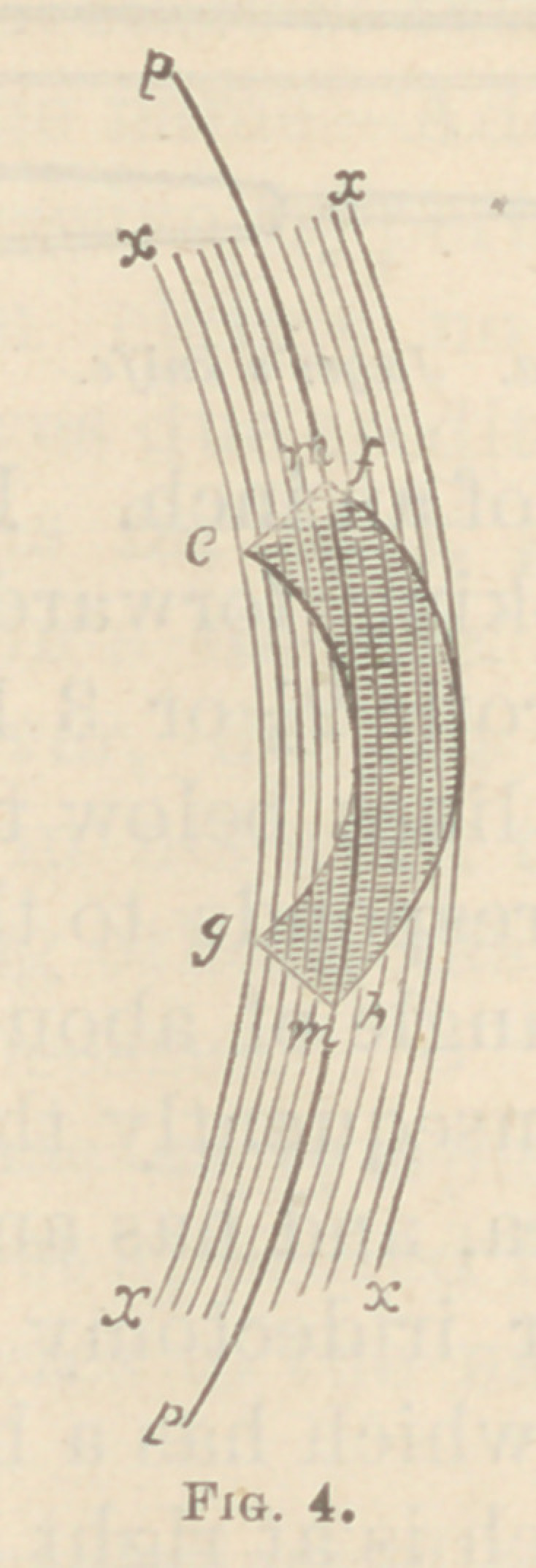


**Fig. 5. f5:**
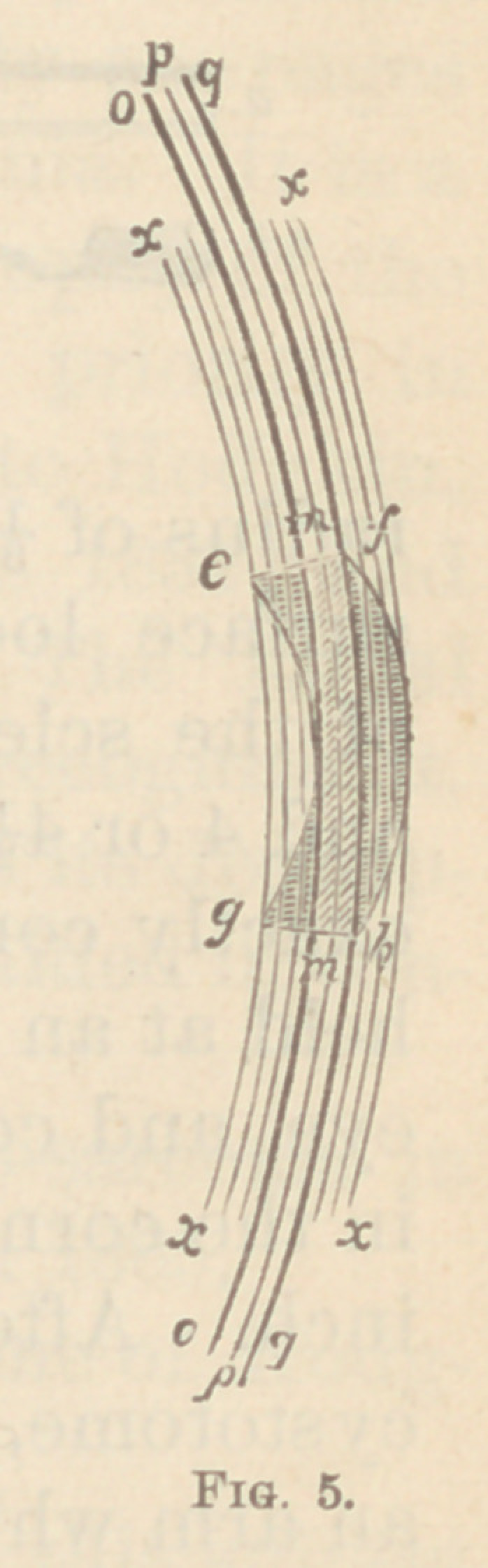


**Figure f6:**